# Approach to the Poisoned Patient

**DOI:** 10.21980/J8264S

**Published:** 2021-01-15

**Authors:** Kennon Heard, Matthew Zuckerman

**Affiliations:** * University of Colorado Anschutz Medical Campus, Department of Emergency Medicine, Aurora, CO

## Abstract

**Audience:**

This video lecture is appropriate for emergency medical providers who are not comfortable with initial assessment of poisoned patients. Ideal learners include medical students and residents.

**Introduction:**

Poisoning is the leading cause of injury-related mortality in the United States, with more than 40,000 deaths annually.^1^ Of all ED injury-related visits, 2.4% are related to poisoning.^2^ While providers may have access to support services (poison control center, medical toxicology consulting service), they are often responsible for the initial evaluation of poisoned patients. While a large portion of poisoned patients have good outcomes regardless of the care they receive, inappropriate risk assessment and lack of knowledge about basic interventions can put patients at risk. A standardized approach as outlined in the video will help evaluate and treat the vast majority of poisoned patients.

**Educational Objectives:**

By the end of the lecture, learners should be able to: 1) initiate the evaluation of a poisoned patient, 2) identify key interventions to support airway, breathing, and circulation, 3) identify the three components of risk assessment in the poisoned patient, 4) list the four options for gastric decontamination, and 5) select standard diagnostic labs and tests commonly used in evaluating poisoned patients.

**Educational Methods:**

This is an enhanced live action greenscreen recording with video animation.

**Research Methods:**

This video has been made available for a variety of learners on social medial (Facebook and YouTube) and written feedback has been collected on YouTube.

**Results:**

The video has been seen over three thousand times and has received positive comments on social media (Facebook, YouTube). Comments include “dropping some tox knowledge bombs, proving yet again that #ToxRocks,” and “This is an excellent talk, thank you very much, but I think it would be worth mentioning antidotes.”

**Discussion:**

The goal is to expose learners to basic concepts in approaching poisoned patients and focusses on cognitive knowledge and diagnostic frameworks. The learner may watch the on-demand video as part of a flipped classroom experience with clinical cases, or as a just-in-time resource before seeing a patient who has been poisoned.

**Topics:**

Poisoning, resuscitation, risk assessment, gastric decontamination, occult ingestion, supportive care.

## USER GUIDE

**Table:** 

List of Resources:	
Abstract	1
User Guide	3
Approach to the Poisoned Patient Video	4

**Learner Audience:**

Medical students, Interns, Junior Residents

**Time Required for Implementation:** 18 minutes

**Topics:**

Poisoning, resuscitation, risk assessment, gastric decontamination, occult ingestion, supportive care.

**Objectives:**

By the end of the lecture, learners should be able to:

1. Initiate the evaluation of a poisoned patient.
2. Identify key interventions to support airway, breathing, and circulation.
3. Identify the three components of risk assessment in the poisoned patient.
4. List the four options for gastric decontamination.
5. Select standard diagnostic labs and tests commonly used in evaluating poisoned patients.

### Linked objectives and methods

The goal is to expose learners to basic concepts in approaching poisoned patients and focusses on cognitive knowledge and diagnostic frameworks. The learner may watch the on-demand video as part of a flipped classroom experience with clinical cases, or as a just-in-time resource before seeing a patient who has been poisoned. Alternative formats such as live lecture or high-fidelity simulation would not provide asynchronous education. In this format, the lecturer first outlines an overview to evaluation of the poisoned patient (objective 1). He then begins to discuss the key interventions for resuscitation at approximately 1 minute (objective 2). This includes airway interventions as well as approach to dysrhythmias. He discusses the three components of risk assessment at approximately 7.5 minutes (objective 3). Specifically this includes considering the drug, the dose, and the patient. He moves on to discuss risk mitigation, initially explaining gastric decontamination at approximately 11 minutes (objective 4). He outlines an approach to testing for occult ingestion at approximately 13.5 minutes (objective 5). Finally, he discusses disposition and observation duration at approximately 15 minutes. He reviews all of his recommendations in closing at approximately 17 minutes.

### Link to Lecture

- https://youtu.be/JSx86YCGFgU

### Results and tips for successful implementation

This can be used as a tool for flipped classroom learning or in a clinical setting just-in-time resource before seeing a poisoned patient. The video has been seen over three-thousand times and has received positive comments on social media (Facebook, YouTube). Comments include “dropping some tox knowledge bombs, proving yet again that #ToxRocks,” and “This is an excellent talk, thank you very much, but I think it would be worth mentioning antidotes.” We have added mention of antidotes as well as discussion of naloxone for opioid toxicity as a result of feedback. While we have not received formal feedback from on-site learners, the video has been liked/retweeted/shared over 50 times. YouTube audience retention metrics suggest that average view duration was over 6 minutes, and relative audience retention was above average compared to YouTube videos of similar lengths.

### Technology necessary

The video can be streamed on any electronic screen with access to the internet and is appropriate for computer or mobile phone screens. Approximately 61% and 33% of YouTube viewers watched on computer and mobile devices, respectively.

## Supplementary Information



## Figures and Tables

**Figure:**
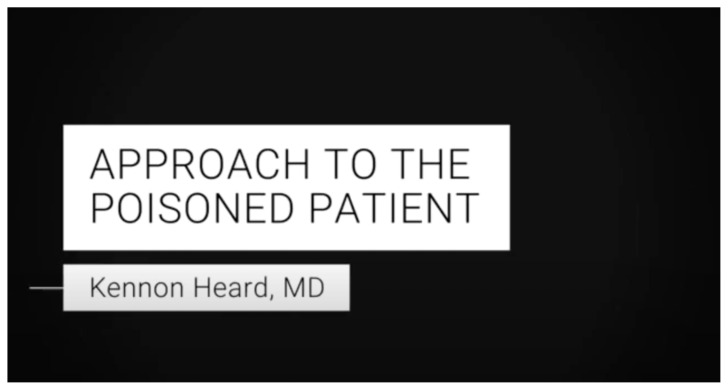
Please see associated video file Video Link: https://youtu.be/JSx86YCGFgU
